# Relationships between care burden, resilience, and depressive symptoms among the main family caregivers of stroke patients: A cross-sectional study

**DOI:** 10.3389/fpsyt.2022.960830

**Published:** 2022-09-20

**Authors:** Linlin Fang, Mengyuan Dong, Wenbo Fang, Jin Zheng

**Affiliations:** ^1^Department of Nursing, The First Affiliated Hospital of China Medical University, Shenyang, China; ^2^School of Nursing and Rehabilitation, Shandong University, Jinan, China; ^3^School of Civil and Hydraulic Engineering, Tibet Agriculture and Animal Husbandry University, Linzhi, China

**Keywords:** care burden, resilience, depressive symptoms, stroke, family caregivers

## Abstract

**Objectives:**

This study aims to explore the potential mediating role of resilience between care burden and depressive symptoms in family caregivers of stroke patients.

**Methods:**

A cross-sectional study was conducted with a convenience sample involving 245 main family caregivers of stroke patients recruited from the neurology department of a Tertiary A hospital in China. Mediation analyses were conducted using the PROCESS macro (Model 4) for SPSS, applying the Bootstrap analysis with 5,000 samples and a 95% confidence interval.

**Results:**

The results showed that with constant hemiplegia side, Barthel Index, education level, monthly income, care time per day, and living with patients in regression equations, the resilience partially mediated the correlation of care burden and depressive symptoms with a mediation effect ratio of 26.32%.

**Conclusions:**

Resilience plays a mediating role in the correlation between care burden and depressive symptoms.

**Impact:**

The findings indicated a protective effect of resilience in alleviating the negative influences of care burden on depressive symptoms, suggesting that resilience-training intervention may be developed to mitigate depressive symptoms of the main family caregivers of stroke patients.

## Introduction

Stroke is one of the leading causes of adult disability and mortality globally (1, 2), particularly in China (3). Currently, stroke survivors have to cope with severe physical, cognitive, and emotional impairments. Indeed, over two-thirds of stroke survivors require assistance in daily life (4). However, due to the limited community health service, and heavy economic burden, most patients choose to be rehabilitated at home with family caregivers providing care after discharge, and care is provided by family caregivers (5). Nevertheless, family caregivers are experiencing difficulties in employment, finance, sleeping, leisure activities (6), and social activities (7), resulting in degraded life quality, and physical and mental health (8, 9). The care burden involves physical, psychological, and social disruption related to the negative caring experience, which can be divided into objective and subjective components (10). It has been reported that 68.4% of the caregivers of stroke patients in China had a moderate burden and above burden (11), indicating that care burden is a severe issue for caregivers.

Family caregivers are facing huge financial burdens, social pressure, and mental distress (12). A previous study reported a high incidence of negative emotions in caregivers, including low satisfaction with leisure time (13), loss of happiness, loneliness, depression, and a sense of imprisonment (14). Indeed, 53.9% of the caregivers of stroke patients in China have varying degrees of depressive symptoms (11), which might be related to the care burden of family caregivers of stroke patients. Heavy burden leads to emotional exhaustion of caregivers and reduces their enthusiasm, thus affecting the quality of care provided (15). Previous studies showed that caregivers with depressive symptoms were more likely to increase the risk of patients' depressive symptoms (16) and even increased the odds of 6-month mortality of stroke survivors (17). However, some caregivers with care burdens do not experience depression, which emphasizes the essential role of protective factors, such as resilience. Resilience is defined as the ability to effectively adapt to trauma and/or adversity (18). Previous studies have shown that people with higher resilience would actively cope with adversity and rapidly adapt to changes (19, 20). Meanwhile, resilience partially mediates the correlation between negative life events and the mental health of caregivers of patients with advanced cancers (21) and diabetes (22). However, few studies have explicitly tested the mediating effects of resilience between care burden and depressive symptoms of family caregivers of stroke patients.

According to previous studies, care burden is correlated with depressive symptoms, and this correlation can be mediated by resilience. Therefore, it is assumed in this study that there is a correlation between care burden and depressive symptoms, and this relationship might be mediated by the resilience of family caregivers of stroke patients.

## Materials and methods

### Study design and participants

In this study, a cross-sectional study and a convenient sampling method were employed. The participants were caregivers of patients admitted to the neurology department ward of a Tertiary A hospital in Shenyang, China during 6 January−20 July 2021. An ethical counsel permit (Ref. 402/2020 on 4 January 2021) was issued by the Medical Ethical Committee of the First Affiliated Hospital of China Medical University, and informed consent was obtained from all participants under the Helsinki Accords.

#### Inclusion criteria

Patients: (1) the patients met the diagnostic criteria of the 4th National Cerebrovascular Disease (The 4th National Symposium on Cardiovascular Disease of the Chinese Medical Association, 1996). Classification of stroke was confirmed by brain computed tomography or magnetic resonance imaging; (2) the score of the Barthel Index ≤95.

Family caregivers: (1) 18 years old and above; (2) spent the longest time with the patients per day; (3) unpaid for the care provided; (4) the care time was no <3 months; (5) voluntarily participate in this study.

#### Exclusion criteria

Family caregivers: (1) suffering from one or more stressful life events within the past 2 weeks (e.g., divorce, widowhood, and loss of job); (2) having a severe physical illness, such as malignancy and intellectual-psychiatric issues; (3) incomplete investigation due to communication or reading and writing obstacles.

### Data collection

During January 2021–July 2021, 250 questionnaires were collected from the participants and 245 of them (valid response rate = 98%) were used for data analysis. Five questionnaires were excluded due to data missing. Data collection was completed by a trained researcher using a self-reported questionnaire. The researcher explained the aims of the study to participants and informed them that the collected data will be kept confidential, and that they had the right to refuse participation. If they agree to participate, they will sign a written informed consent. Questionnaires were completed independently by the participant and collected immediately. Additionally, any participant who wished to quit anytime during the study was allowed to do so.

### Measurements

#### Demographic characteristics

The demographic data collected from the patients include gender, age, insurance, stroke subtypes, language barriers, dysphagia, cognitive barriers and hemiplegia side. The demographic data collected from the caregivers include gender, age, education level, monthly income, employment status, relationship with the patient, total care duration, care time per day, and living with patients.

#### The Barthel Index (BI)

The Barthel Index (BI) was developed by Mahoney (23) in 1965 and has been widely employed to assess self-care activities including eating, bathing, grooming, dressing, using the toilet, transferring from bed to chair, walking, stair climbing, bowel control, and bladder control (23). It consists of 10 items and each item is supposed to be scored based on a 5-point Likert scale. The total score ranges from 0 (total dependence) to 100 (total independence), with 0–20 points defining total dependence, 21–60 defining severe dependence, 61–90 defining moderate dependence, 91–99 defining slight dependence, and 100 defining total independence (23, 24). The validity and reliability of this tool for use in the Chinese elderly population have been well-established (25). In this study, the Cronbach's alpha value was 0.87.

#### Zarit caregiver burden interview (ZBI)

The Chinese version of the ZBI scale (26), which was used to measure caregivers' perceived burden of providing informal care (27). The scale consists of 22-items assessing role strain and personal strain (28), and each item is supposed to be scored based on a 5-point Likert scale (0 = never, 1 = seldom, 2 = sometimes, 3 = often, and 4 = almost always) (29). The total score ranges from 0 to 88, with 0–20 points defining negligible or no load, 21–40 defining intermediate load, 41–60 defining large load, and 61–88 defining excessive load (30). The Chinese version of ZBI has satisfactory psychometric properties (31). In this study, the Cronbach's alpha value was 0.93.

#### Connor-Davidson resilience scale (CD-RISC)

The CD-RISC scale was originally developed by Connor and Davidson (32) and translated into Chinese by Yu and Zhang (33), is one of the most widely used scales to measure resilience. The scale consists of 25-items assessing tenacity, strength and optimism (33), and each item is supposed to be scored based on a 5-point Likert scale from 0 (not true at all) to 4 (true nearly all the time) (32). The total score ranges from 0 to 100 and the score is proportional to the resilience level (32). The Chinese version of CD-RISC exhibited good reliability and validity (34). In this study, the Cronbach's alpha value was 0.94.

#### Center for epidemiological survey depression scale (CES-D)

The Chinese version of the CES-D scale was designed to evaluate the depressive symptoms and risk of disorder in a non-psychiatric person (35). The scale consists of 20-items assessing depressed feelings, somatic complaints, positive feelings and international relationships, and each item is supposed to be scored based on a 4-point Likert scale from 0 (rarely or none of the time) to 3 (most of the time) (35). The total score ranges from 0 to 60 and a score ≥ 16 indicates an elevated level of depressive symptoms (35). Additionally, a score of 16–23 and ≥24 were classified as moderate and severe depressive symptomatology (36). The Chinese version of CES-D has been widely used in China with good reliability and validity (37). In this study, the Cronbach's alpha value was 0.95.

### Statistical analysis

Statistical analyses were conducted utilizing SPSS version 26.0. Normal distribution tests were verified by using Kolmogorov-Smirnov and Shapiro-Wilk statistics. Continuous variables were presented as mean ± standard deviation (*SD*), whereas classification variables were presented as frequency and percentages (%). Independent sample *t*-test or single-factor variance was conducted to identify differences in depressive symptoms concerning the characteristics of caregivers and stroke survivors. Pearson's correlation analysis was employed to explore the correlation between care burden, resilience, and depressive symptoms. The mediation model was analyzed using Model 4 in the PROCESS Marco (38) version 3.3 with 5,000 iteration bootstrapping to measure the indirect effect and 95% confidence intervals (*CI*) were estimated. Parameters of indirect effects were considered statistically significant when the 95% *CI* did not include 0(39). Hemiplegia side, education level, monthly income, living with patients, care time per day, and the BI score were included as covariates since these variables exhibited significant differences in depressive symptoms and were significantly associated with depressive symptoms. A two-sided *p* < 0.05 was considered statistically significant.

## Results

### Sociodemographic characteristics

As shown in Table 1, stroke survivors ranged have ages from 34 to 89 years old (mean = 64.09, *SD* = 9.66), 66.53% of them were males and 90.20% of them needed help for daily activities. Of the caregivers aged 27–80 years old (mean = 59.05, *SD* = 1.00), 78.78% of them were females and 75.10% of them were the spouse of the patient. 20.00, 67.35, and 12.65% has a total score (BI) of 0–60, 61–90, and 91–99, respectively.

**Table 1:** Descriptive statistics for demographic characteristics and differences in depressive symptoms (*N* = 245).

**Variable**	***N***	**%**	**Depressive symptoms**	
			***M ±SD***	***F* or *t* (*P*)**
**Patients**				
Gender				1.398 (0.163)
Male	163	66.53	22.96 ± 10.21	
Female	82	33.47	21.10 ± 9.01	
Age (years)				1.796 (0.149)
<55	45	18.37	23.71 ± 11.45	
55–64	70	28.57	23.79 ± 9.42	
65–74	105	42.86	21.46 ± 9.71	
≥75	24	9.80	19.48 ± 7.74	
Health insurance				−0.781 (0.436)
Yes	232	94.69	22.22 ± 9.94	
No	12	4.90	24.50 ± 7.79	
Stroke subtypes				1.210 (0.300)
Ischemic stroke	204	83.33	22.40 ± 10.14	
Hemorrhagic stroke	18	7.35	24.72 ± 8.24	
Both	23	9.39	20.00 ± 8.06	
Language barriers				1.776 (0.184)
Yes	77	31.43	23.31 ± 10.22	
No	168	68.57	21.89 ± 9.67	
Dysphagia				1.960 (0.163)
Yes	23	9.39	26.30 ± 8.44	
No	222	90.61	21.92 ± 9.91	
Cognitive barriers				0.422 (0.517)
Yes	10	4.08	21.40 ± 9.24	
No	235	95.92	22.37 ± 9.89	
Hemiplegia side				2.991 (<0.050)
None	11	4.49	20.64 ± 10.24	
Left	108	44.08	21.22 ± 9.14	
Right	74	30.20	21.72 ± 10.34	
Both	52	21.22	25.88 ± 9.91	
Total scores (BI)				8.882 (<0.001)
0–60	49	20.00	27.47 ± 10.84	
61–90	165	67.35	21.05 ± 9.48	
91–99	31	12.65	21.03 ± 7.42	
**Caregivers**				
Gender				−0.752 (0.453)
Males	52	21.22	21.42 ± 8.82	
Females	193	78.78	22.58 ± 10.11	
Age				0.231 (0.875)
<55	83	33.88	21.96 ± 9.59	
55–64	77	31.43	22.36 ± 9.50	
65–74	71	28.98	23.00 ± 10.89	
≥75	14	5.71	21.00 ± 8.21	
Education status				8.641 (<0.001)
Primary school at most	50	20.41	27.08 ± 10.46	
Junior high school	80	32.65	23.63 ± 9.42	
High school/technical school	80	32.65	19.71 ± 8.19	
College and above	35	14.29	18.60 ± 10.42	
Monthly income (RMB, yuan)				10.811 (<0.001)
<2,500	58	23.67	27.93 ± 10.14	
2,500–3,500	93	37.96	21.72 ± 8.76	
3,500–4,500	70	28.57	20.33 ± 9.17	
>4,500	24	9.80	17.04 ± 9.61	
Working status				4.489 (<0.050)
Employed	91	37.14	22.54 ± 9.60	
Unemployed	60	24.49	25.10 ± 10.87	
Retired	93	37.96	20.32 ± 8.99	
Relationship with patient				5.843 (<0.001)
Spouse	184	75.10	22.55 ± 9.58	
Offspring	52	21.22	20.31 ± 9.48	
Parents	5	2.04	38.60 ± 10.90	
Sibling	4	1.63	18.50 ± 7.94	
Duration of care time (month)				0.677 (0.567)
3–6	89	36.33	21.34 ± 9.68	
6–12	45	18.37	22.58 ± 8.50	
12–36	52	21.22	22.31 ± 9.96	
>36	59	24.08	23.68 ± 10.96	
Care time per day (hours)				7.086 (<0.001)
<4	102	41.63	19.30 ± 7.54	
4–8	98	40.00	23.53 ± 10.16	
8–16	34	13.88	26.21 ± 11.79	
>16	11	4.49	27.82 ± 11.75	
Living with patients				2.407 (<0.050)
Yes	232	94.69	22.69 ± 9.78	
No	13	5.31	16.00 ± 9.22	

SD, standard deviation; BI, the Barthel Index.

### Descriptive and correlative analysis

The average scores of care burden, resilience, and depressive symptoms of caregivers were 43.89 ± 13.40, 55.68 ± 11.01, and 22.33 ± 9.85, respectively. 72.65% of the caregivers had depressive symptoms. The results of Pearson's correlation analysis revealed that care burden was positively associated with depressive symptoms (*r* = 0.58, *p* < 0.01). Additionally, resilience was negatively associated with care burden (*r* = −0.26, *p* < 0.01) and depressive symptoms (*r* = −0.70, *p* < 0.01), as shown in Table 2.

**Table 2:** Means, standard deviations, and correlations among variables.

**Variable**	***M ±SD***	**1**	**2**	**3**
1. Care burden	43.89 ± 13.40	–		
2. Resilience	55.68 ± 11.01	−0.26**	–	
3. Depressive symptoms	22.33 ± 9.85	0.58**	−0.70**	–

^**^p < 0.01.

### Mediating effect of resilience

**Figure 1:**
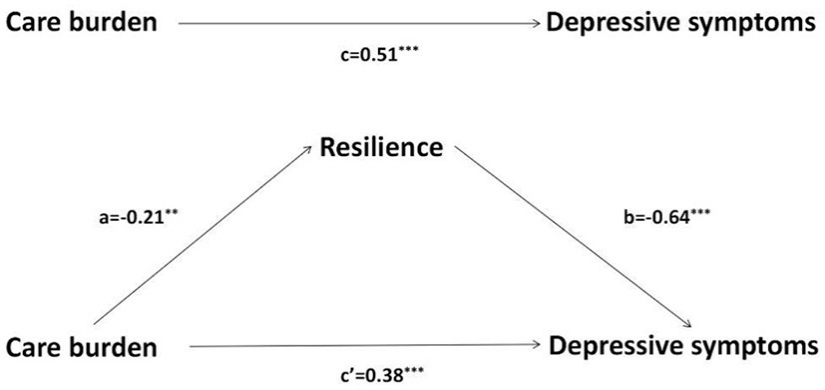
The mediating effects of resilience between care burden and depressive symptoms. **p < 0.01, ***p < 0.001. Standardized regression coefficient for the mediation model. **(a)** is the effect of care burden on resilience, **(b)** is the effect of resilience on depressive symptoms, **(c)** is the total effect of care burden on depressive symptoms, **(c')** is the direct effect of care burden on depressive symptoms (15).

To verify the proposed hypothesis conceptual model, Model 4 of Hayes' PROCESS macro was applied to establish three regression models (see Table 3 and Figure 1). After controlling the hemiplegia side, BI score, education level, monthly income, care time per day, and living with patients, the care burden was positively associated with depressive symptoms (β = 0.51, *p* < 0.001). After adding resilience, the positive association between care burden and depressive symptoms remained significant (β = 0.38, *p* < 0.001), while resilience was negatively correlated with care burden (β = −0.21, *p* < 0.01) and depressive symptoms (β = −0.64, *p* < 0.001).

**Table 3:** Mediating effect of resilience between care burden and depressive symptoms.

**Controls**	**Model 1 (resilience)**			**Model 2 (depressive symptoms)**			**Model 3 (depressive symptoms)**		
	**β**	***SE***	***t***	**β**	***SE***	***t***	**β**	***SE***	***t***
Hemiplegia side	0.04	0.70	0.65	0.06	0.60	1.13	0.08	0.44	2.12*
The Barthel Index	−0.17	1.38	−2.37**	0.06	1.17	0.96	−0.04	0.88	−0.84
Education level	0.03	0.87	0.40	−0.03	0.74	−0.47	−0.01	0.55	−0.27
Monthly income	0.48	0.90	6.31***	−0.21	0.76	−2.89**	0.10	0.61	1.68
Care time per day	−0.17	0.92	−2.38	0.09	0.78	1.32	−0.02	0.59	−0.37
Living with patients	−0.10	2.72	−1.88	−0.04	2.30	−0.75	−0.11	1.72	−2.69**
Independence variable									
Care burden	−0.21	0.05	−3.37**	0.51	0.04	8.82***	0.38	0.03	8.63***
Mediator									
Resilience							−0.64	0.04	−13.93***
R^2^	0.33			0.40			0.67
*F*	16.91***			22.67***			60.25***		

^*^p < 0.05, ^**^p < 0.01, ^***^p < 0.001. Bootstrap sample size = 5,000. β, standardized coefficients; SE, Standard Error; t, t-test value; F, F-test value; R^2^, explanatory power.

Then, bootstrapping was executed to determine the statistical significance of the mediating effect of resilience. We adopted the method of random sampling to extract 5000 Bootstrap samples from the original data (*N* = 245). The results demonstrated that the total effect of care burden on depressive symptoms was 0.38 [95% *CI* (0.29–0.46)], with the direct effect and the indirect effect being 0.28 [95% *CI* (0.22–0.35)] and 0.10 [95% *CI* (0.04–0.16)], respectively. The 95% *CI* did not contain 0, indicating that resilience played a mediating role in the correlation of care burden and depressive symptoms, with a mediating contribution rate of 26.32% (0.10/0.38), as shown in Table 4.

**Table 4:** Bootstrap analysis of mediation effect significance test (*N* = 245).

**Effect**	***Effect***	***SE***	**95%** ***CI***	
			***LLCL***	***UICL***
Total effect	0.38	0.04	0.29	0.46
Direct effect	0.28	0.03	0.22	0.35
Indirect effect	0.10	0.03	0.04	0.16

Bootstrap sample size = 5,000. SE, Standard Error; CI, Confidence Interval; LLCL, Lower Limit Confidence Interval; ULCL, Upper Limit Confidence Interval.

## Discussion

The study aims to clarify the correlation between care burden and depressive symptoms by using the Kumpfer's resilience model. First, the mean score of depressive symptoms was 22.33 ± 9.85, which was higher than that reported in previous studies using the same tool for caregivers of patients with other diseases, including dementia (40) and cancer (41). The difference can be attributed to stroke-related disabilities and long disease duration (42), which poses a heavy burden on caregivers of stroke patients.

As the period of data collection in this study coincided with the COVID-19 pandemic, the incidence of depressive symptoms (72.65%) among caregivers was different from those in previous studies (40–71%) (43, 44). The uncertainty induced by the epidemic would increase the psychological pressure [e.g., infection risk (45), unemployment, financial insecurity (46)] on everyone, including caregivers enrolled in this study. Specifically, the decrease of social interaction could have a negative impact on mental health, since caregivers' life and normal rest may get irregular due to restrictions on outdoor activities (47). COVID-19-related financial distress and work impairment were also associated with higher symptom levels of depression (48). A recent study (49) showed that 78.5% of caregivers of children with kidney diseases reported depressive symptoms during the pandemic, which was 32.8–48.3% higher than those in previous studies (50, 51). Meanwhile, the incidence of subjective depressive symptoms increased from 5.9 to 60% among caregivers of patients with dementia (52). On the other hand, patient caring is more challenging due to the restriction of hospitalizations and the complicated admissions process during the pandemic (53). Another recent study reported a 40% drop in stroke admissions (54), and it complained that strict measures due to the pandemic can lead to increased anxiety and distress (55).

There were statistically significant differences in the hemiplegia side, the BI and education, monthly income, working status, correlation with patients, care time per day, and living with patients among the depressive symptoms of the caregivers. Similar to previous studies (11, 16), depressive symptoms are associated with the severity of functional disability of the patients as they are more likely to rely on caregivers for support and care (56). Meanwhile, caregivers with a higher education level tend to experience fewer depressive symptoms, which may be attributed to better ways to insight into illness and seek help (57). The study has shown that unemployment and low income are risk factors for depressive symptoms as limited economic resources, substantial uncertainty and income volatility expose them to physical and mental stress (58). Additionally, the length of care time was proportional to depression. This may be attributed to the fact that a long care time would let to more disruptions in daily life, causing increased stress levels (59). In some studies, parental caregivers exhibited more depressive symptoms compared with spousal caregivers as they are more vulnerable due to physical limitations (60) and prone to have negative emotions related to the future they had envisioned for the child [e.g., care for the child after their death (61)]. Nevertheless, some studies stated that spouses exhibited more depressive symptoms (62, 63), which may be attributed to the fact that spousal caregivers tend to be overwhelmed by conflicting demands such as work, children, and household chores (5). The result indicates that healthcare workers should focus on spousal and parental caregivers with low income, low education, unemployment, living with patients and long-term care in future work, and develop appropriate interventions to reduce depressive symptoms and improve the life quality of both stroke patient and his/her family caregivers.

Notably, the results of the present study showed that there is a statistically significant and inverse relationship between care burden and depressive symptoms in family caregivers. Our findings support previous research (64, 65) suggesting that caregivers who score high care burden have also high depression. Despite these similar findings in the literature, we don't see a ready explanation for this association. In future research we need to further examine the dynamic mechanisms between care burden and depressive symptoms of caregivers. Also, our results showed that resilience could partly mediate the relationship between care burden and depressive symptoms. This is consistent with previous studies that examined resilience as a possible mediator (66), including in the context of COVID-19 research (67). One possible explanation may be that people with a lower level of resilience tend to negatively confront adversity in unhealthy ways, such as mood disturbances, persisting fatigue, and sleep changes (68). Specifically, the psychobiological mechanisms underlying resilience has shown that resilience had a relation to neurochemical, neuropeptide, and hormonal when the response to stressful things (69, 70), people with higher resilience tend to reduce psychobiological allostatic load, and balance neural systems, which could maintain normal psychological function and thus can confront stress actively (71). Besides, individuals with higher resilience are better at coping with stressful events, they tend to make active attempts to adjust the relationship between the environment and individuals, make full use of various resources, and achieve a good state of adaptation (72, 73). Therefore, resilience seemed to be one of the possible mechanisms to resist mental disorders who exposed to care burden, which confirmed Kumpfer's resilience model.

Although family caregivers are often critical to maintain the patients' health, there has been little emphasis on how clinicians should relate to family caregivers (74). Caregivers become “the invisible patient” and often feel tense and upset (74). Hence there is a need to undertake necessary precautions to protect their health. Among patients, a handful of resilience-based interventions have shown promising results for outcomes such as resilience, stress, and anxiety (75, 76). However, there are few published recommendations for conducting intervention trials with stroke caregivers. Some recent studies suggests that a strength-oriented psychoeducational program can reduce depressive symptoms and improved life changes for caregivers (77), as well as the assessment of the risk factors of depressive symptoms (78). The current study suggests that we should assess the situation of care burden and depressive symptoms of caregivers, screen for its main influencing factors, and take effective programs such as social and financial support (79), increased post-traumatic, better patient-caregiver relationships, growth improvement in the competence and self-esteem of caregivers (80). In addition, resilience plays an important role for caregivers' mental health also means it is possible to alleviate the depressive symptoms of caregivers by promoting the level of resilience. Specifically, social support is one of the important sources for the development of resilience, which may ultimately help lessen depressive symptoms (81). Self-compassion and mindfulness training are also related to higher resilience (82). Moreover, some research has shown that love for family, feeling responsible toward the family (83), ability to analyze the current situation, and capability to establish relationships (84) are some of the motivations for resilience. In addition to the aforementioned approach, Overall, the sources of strength can provide intervention targets for promoting resilience and care burden, and thus alleviate the depressive symptoms. Insufficient evidence is available to show that psychoeducational interventions should be implemented in the families of stroke survivors.

There are some limitations in our study to be considered. Firstly, this study is a cross-sectional study, and it is difficult to determine the causal connections between the variables. Therefore, future studies can use longitudinal research to explore the causal relationship between variables. Secondly, we used a self-rating questionnaire for screening for depressive symptoms instead of a clinical diagnosis from psychiatrists. Irrespective, the instrument is a validated depressive symptoms screening tool. Thirdly, our study focused only on the association between care burden, resilience, and depressive symptoms. Further investigation needs to be taken into consideration to explore other social psychology and emotional predictors for the level of depressive symptoms in caregivers of stroke survivors, such as society, family environment factors, and so on. Finally, the COVID-19 level of psychological distress in the current sample has not been assessed, the results must be interpreted with caution. However, in large samples, the current study adds valuable information to incipient efforts to understand care burden and its consequences for family caregivers of patients with stroke, it can help to provide first insights into the research field and help to define directions for the future.

## Conclusions

The correlation of care burden, resilience and depressive symptoms in the main family caregivers of stroke patients was explored. The results showed a severe mental health burden on the main family caregivers, especially spousal and parental caregivers, of stroke patients. The self-care ability of patients and conditions of caregivers (e.g., education, income, employment, relationship with the patient, care time per day, and living with patients) were had a direct correlation with depressive symptoms. Care burden was positively correlated with depressive symptoms, while the mediating effect of resilience helps to alleviate depressive symptoms of caregivers with high care burden. This study facilitates understanding and prompt assessment of mental health of the main family caregivers of stroke patients, and the development of resilience-promoting measures in the health care system.

## Data availability statement

The raw data supporting the conclusions of this article will be made available by the authors, without undue reservation.

## Ethics statement

Ethical counsel permit (Approval number: 402/2020) was approved by the Medical Ethical Committee of the First Affiliated Hospital of China Medical University, and informed consent was received from each participant. All procedures performed in the study involving human participants were in accordance with the ethical standards of the hospital, the National Research Committee, and the 1964 Helsinki Declaration (as revised in Brazil 2013).

## Author contributions

LF and JZ conceptualized and designed the study. LF collected the data and prepared Figure 1 and Tables 1–4. LF and MD analyzed the data. JZ reviewed the analyses. LF, WF, and MD drafted the initial version of the manuscript. All authors contributed to revising, editing, and finalizing the manuscript. All authors have read and agreed to the published version of the manuscript.

## Conflict of interest

The authors declare that the research was conducted in the absence of any commercial or financial relationships that could be construed as a potential conflict of interest.

## Publisher's note

All claims expressed in this article are solely those of the authors and do not necessarily represent those of their affiliated organizations, or those of the publisher, the editors and the reviewers. Any product that may be evaluated in this article, or claim that may be made by its manufacturer, is not guaranteed or endorsed by the publisher.

## References

[1] Rajsic S Gothe H Borba HH Sroczynski G Vujicic J Toell T. Economic burden of stroke: a systematic review on post-stroke care. Eur J Health Econ. (2019) 20:107–34. 10.1007/s10198-018-0984-0 29909569

[2] Vos T Lim SS Abbafati C Abbas KM Abbasi M Abbasifard M. Global burden of 369 diseases and injuries in 204 countries and territories, 1990-2019: a systematic analysis for the Global Burden of Disease Study 2019. Lancet. (2020) 396:1204–22. 10.1016/S0140-6736(20)30925-9 33069326 PMC7567026

[3] Krishnamurthi RV Ikeda T Feigin VL. Global regional and country-specific burden of ischaemic stroke, intracerebral haemorrhage and subarachnoid haemorrhage: a systematic analysis of the global burden of disease study 2017. Neuroepidemiology. (2020) 54:171–9. 10.1159/000506396 32079017

[4] Winstein CJ Stein J Arena R Bates B Cherney LR Cramer SC. Guidelines for adult stroke rehabilitation and recovery: a guideline for healthcare professionals from the American Heart Association/American Stroke Association. Stroke. (2016) 47:e98–169. 10.1161/STR.0000000000000098 27145936

[5] Lu Q Mårtensson J Zhao Y Johansson L. Living on the edge: family caregivers' experiences of caring for post-stroke family members in China: a qualitative study. Int J Nurs Stud. (2019) 94:1–8. 10.1016/j.ijnurstu.2019.02.016 30928717

[6] Caro CC Costa JD Da Cruz DMC. Burden and quality of life of family caregivers of stroke patients. Occup Ther Health Care. (2018) 32:154–71. 10.1080/07380577.2018.1449046 29578827

[7] Batuecas-Caletrío J Rodríguez-Martín B. Family and personal coping process after a haemorrhagic stroke, a life-history. Curr Psychol. (2020) 1–10. 10.1007/s12144-020-01204-2

[8] Kruithof WJ Post MW Van Mierlo ML Van Den Bos GA De Man-Van Ginkel JM Visser-Meily JM. Caregiver burden and emotional problems in partners of stroke patients at two months and one year post-stroke: determinants and prediction. Patient Educ Couns. (2016) 99:1632–40. 10.1016/j.pec.2016.04.007 27103190

[9] Albayrak I Biber A Çalişkan A Levendoglu F. Assessment of pain, care burden, depression level, sleep quality, fatigue and quality of life in the mothers of children with cerebral palsy. J Child Health Care. (2019) 23:483–94. 10.1177/1367493519864751 31319696

[10] Caserta MS Lund DA Wright SD. Exploring the Caregiver Burden Inventory (CBI): further evidence for a multidimensional view of burden. Int J Aging Human Dev. (1996) 43:21–34. 10.2190/2DKF-292P-A53W-W0A8 8886874

[11] Hu P Yang Q Kong L Hu L Zeng L. Relationship between the anxiety/depression and care burden of the major caregiver of stroke patients. Medicine. (2018) 97:e12638. 10.1097/MD.0000000000012638 30290641 PMC6200450

[12] Ashghali Farahani M Najafi Ghezeljeh T Haghani S Alazmani-Noodeh F. The effect of a supportive home care program on caregiver burden with stroke patients in Iran: an experimental study. BMC Health Serv Res. (2021) 21:346. 10.1186/s12913-021-06340-4 33858400 PMC8048267

[13] Pendergrass A Hautzinger M Elliott TR Schilling O Becker C Pfeiffer K. Family caregiver adjustment and stroke survivor impairment: a path analytic model. Rehabil Psychol. (2017) 62:81–8. 10.1037/rep0000118 28165262

[14] Seal K Murray CD Seddon L. The experience of being an informal “carer” for a person with cancer: a meta-synthesis of qualitative studies. Palliat Support Care. (2015) 13:493–504. 10.1017/S1478951513001132 24606765

[15] An Y Fu G Yuan G. Quality of life in patients with breast cancer: the influence of family caregiver's burden and the mediation of patient's anxiety and depression. J Nerv Ment Dis. (2019) 207:921–6. 10.1097/NMD.0000000000001040 31517713

[16] Malhotra R Chei CL Menon E Chow WL Quah S Chan A. Short-term trajectories of depressive symptoms in stroke survivors and their family caregivers. J Stroke Cerebrovasc Dis. (2016) 25:172–81. 10.1016/j.jstrokecerebrovasdis.2015.09.012 26476585

[17] Zhao J Zeng Z Yu J Xu J Chen P Chen Y. Effect of main family caregiver's anxiety and depression on mortality of patients with moderate-severe stroke. Sci Rep. (2021) 11:2747. 10.1038/s41598-021-81596-8 33531519 PMC7854741

[18] Luthar SS Cicchetti D Becker B. The construct of resilience: a critical evaluation and guidelines for future work. Child Dev. (2000) 71:543–62. 10.1111/1467-8624.00164 10953923 PMC1885202

[19] Sheerin CM Lind MJ Brown EA Gardner CO Kendler KS Amstadter AB. The impact of resilience and subsequent stressful life events on MDD and GAD. Depress Anxiety. (2018) 35:140–7. 10.1002/da.22700 29172241 PMC5794521

[20] Bhatnagar S. Rethinking stress resilience. Trends Neurosci. (2021) 44:936–45. 10.1016/j.tins.2021.09.005 34711401 PMC8616827

[21] Dionne-Odom JN Azuero A Taylor RA Wells RD Hendricks BA Bechthold AC. Resilience, preparedness, and distress among family caregivers of patients with advanced cancer. Support Care Cancer. (2021) 29:6913–20. 10.1007/s00520-021-06265-y 34031751 PMC9733586

[22] Luo D Gu W Bao Y Cai X Lu Y Li R. Resilience outstrips the negative effect of caregiver burden on quality of life among parents of children with type 1 diabetes: an application of Johnson-Neyman analysis. J Clin Nurs. (2021) 30:1884–92. 10.1111/jocn.15739 33656212

[23] Mahoney FI Barthel DW. Functional evaluation: the barthel index. Md State Med J. (1965) 14:61–5. 10.1037/t02366-000 14258950

[24] Shah S Vanclay F Cooper B. Improving the sensitivity of the Barthel index for stroke rehabilitation. J Clin Epidemiol. (1989) 42:703–9. 10.1016/0895-4356(89)90065-6 2760661

[25] Leung SO Chan CC Shah S. Development of a Chinese version of the modified Barthel index– validity and reliability. Clin Rehabil. (2007) 21:912–22. 10.1177/0269215507077286 17981850

[26] Wang L Yang X Hou Z Feng QL. Application and evaluation of Chinese version of zarit caregiver burden interview. Chin J Public Health. (2006) 121:28–32. 10.11847/zgggws2006-22-08-47

[27] Ko KT Yip PK Liu SI Huang CR. Chinese version of the Zarit caregiver burden interview: a validation study. Am J Geriatr Psychiatry. (2008) 16:513–8. 10.1097/JGP.0b013e318167ae5b 18515696

[28] Whitlatch CJ Zarit SH von Eye A. Efficacy of interventions with caregivers: A reanalysis. The Gerontologist. (1991) 31:9–14. 10.1093/geront/31.1.9 2007480

[29] Rankin ED Haut MW Keefover RW Franzen MD. The establishment of clinical cutoffs in measuring caregiver burden in dementia. Gerontologist. (1994) 34:828–32. 10.1093/geront/34.6.828 7843613

[30] Zarit S Zarit J. Instructions for the Burden Interview. University Park, PA: Pennsylvania State University (1987).

[31] Lin CY Wang JD Pai MC Ku LE. Measuring burden in dementia caregivers: confirmatory factor analysis for short forms of the Zarit burden interview. Arch Gerontol Geriatr. (2017) 68:8–13. 10.1016/j.archger.2016.08.005 27580015

[32] Connor KM Davidson JR. Development of a new resilience scale: the Connor-Davidson Resilience Scale (CD-RISC). Depress Anxiety. (2003) 18:76–82. 10.1002/da.10113 12964174

[33] Yu X Zhang J. Factor analysis and psychometric evaluation of the Connor-Davidson Resilience Scale (CD-RISC) with Chinese people. Soc Behav Personal Int J. (2007) 35:19–30. 10.2224/sbp.2007.35.1.1928152997

[34] Wu L Tan Y Liu Y. Factor structure and psychometric evaluation of the Connor-Davidson resilience scale in a new employee population of China. BMC Psychiatry. (2017) 17:49. 10.1186/s12888-017-1219-0 28152997 PMC5290619

[35] Radloff LS. The CES-D scale: a self-report depression scale for research in the general population. Appl Psychol Meas. (1977) 1:385–401. 10.1177/01466216770010030623302475

[36] Rushton JL Forcier M Schectman RM. Epidemiology of depressive symptoms in the National Longitudinal Study of Adolescent Health. J Am Acad Child Adolesc Psychiatry. (2002) 41:199–205. 10.1097/00004583-200202000-00014 11837410

[37] Jiang L Wang Y Zhang Y Li R Wu H Li C. The reliability and validity of the center for epidemiologic studies depression scale (CES-D) for Chinese University Students. Front Psychiatry. (2019) 10:315. 10.3389/fpsyt.2019.00315 31178764 PMC6537885

[38] Baron RM Kenny DA. The moderator-mediator variable distinction in social psychological research: conceptual, strategic, and statistical considerations. J Pers Soc Psychol. (1986) 51:1173–82. 10.1037/0022-3514.51.6.1173 3806354

[39] Lockhart G Mackinnon DP Ohlrich V. Mediation analysis in psychosomatic medicine research. Psychosom Med. (2011) 73:29–43. 10.1097/PSY.0b013e318200a54b 21148809 PMC3366636

[40] Seeher K Low LF Reppermund S Brodaty H. Predictors and outcomes for caregivers of people with mild cognitive impairment: a systematic literature review. Alzheimers Dement. (2013) 9:346–55. 10.1016/j.jalz.2012.01.012 23123229

[41] Jun WH Cha KS Lee KL. The mediating effect of depression on the relationship between social support, spirituality and burnout in family members of patients with cancer. Int J Environ Res Public Health. (2021) 18:1727. 10.3390/ijerph18041727 33578997 PMC7916776

[42] Baumann M Lurbe-Puerto K Alzahouri K Aïach P. Increased residual disability among poststroke survivors and the repercussions for the lives of informal caregivers. Top Stroke Rehabil. (2011) 18:162–71. 10.1310/tsr1802-162 21447466

[43] Shanmugham K Cano MA Elliott TR Davis M. Social problem-solving abilities, relationship satisfaction and depression among family caregivers of stroke survivors. Brain Injury. (2009) 23:92–100. 10.1080/02699050802657020 19191088

[44] Guo Y-L Liu Y-J. Family functioning and depression in primary caregivers of stroke patients in China. Int J Nurs Sci. (2015) 2:184–9. 10.1016/j.ijnss.2015.05.002

[45] Cullen W Gulati G Kelly BD. Mental health in the COVID-19 pandemic. QJM. (2020) 113:311–2. 10.1093/qjmed/hcaa110 32227218 PMC7184387

[46] Shah SMA Mohammad D Qureshi MFH Abbas MZ Aleem S. Prevalence, psychological responses and associated correlates of depression, anxiety and stress in a global population, during the coronavirus disease (COVID-19) pandemic. Commun Ment Health J. (2021)57:101–10. 10.1007/s10597-020-00728-y 33108569 PMC7590908

[47] Okruszek Ł Aniszewska-Stańczuk A Piejka A Wiśniewska M. Zurek K. Safe but lonely? Loneliness, anxiety, and depression symptoms and COVID-19. Front Psychol. (2020) 11:579181. 10.3389/fpsyg.2020.579181 33343454 PMC7747668

[48] Batterham PJ Calear AL Mccallum SM Morse AR Banfield M Farrer LM. Trajectories of depression and anxiety symptoms during the COVID-19 pandemic in a representative Australian adult cohort. Med J Aust. (2021) 214:462–8. 10.5694/mja2.51043 33899939 PMC8207103

[49] Sharma R Jafra BS Tiewsoh K Kumar K Kaur N Sharawat IK. Distress, anxiety, and its correlates among caregivers of children with kidney diseases during COVID-19 pandemic lockdown. Arch Pediatr. (2022) 29:243–8. 10.1016/j.arcped.2022.01.003 35115218 PMC8768425

[50] Willems LM Schubert-Bast S Grau J Hertzberg C Kurlemann G Wiemer-Kruel A. Health-related quality of life in children and adolescents with tuberous sclerosis complex and their caregivers: a multicentre cohort study from Germany. Eur J Paediatr Neurol. (2021) 35:111–22. 10.1016/j.ejpn.2021.10.003 34673401

[51] Gerogianni G Polikandrioti M Alikari V Vasilopoulos G Zartaloudi A Koutelekos I. Factors affecting anxiety and depression in caregivers of hemodialysis patients. Adv Exp Med Biol. (2021) 1337:47–58. 10.1007/978-3-030-78771-4_6 34972890

[52] Bussè C Barnini T Zucca M Rainero I Mozzetta S Zangrossi A. Depression, anxiety and sleep alterations in caregivers of persons with dementia after 1-year of COVID-19 pandemic. Front Psychiatry. (2022) 13:826371. 10.3389/fpsyt.2022.826371 35222125 PMC8866969

[53] Greenberg JA Basapur S Quinn TV Bulger JL Schwartz NH Oh SK. Challenges faced by families of critically ill patients during the first wave of the COVID-19 pandemic. Patient Educ Counsel. (2022) 105:297–303. 10.1016/j.pec.2021.08.029 34507866 PMC8393512

[54] Zhao J Li H Kung D Fisher M Shen Y Liu R. Impact of the COVID-19 epidemic on stroke care and potential solutions. Stroke. (2020) 51:1996–2001. 10.1161/STROKEAHA.120.030225 32432997 PMC7258753

[55] Lee JJ Tsang WN Yang SC Kwok JYY Lou VWQ Lau KK. Qualitative study of chinese stroke caregivers' caregiving experience during the COVID-19 pandemic. Stroke. (2021) 52:1407–14. 10.1161/STROKEAHA.120.032250 33588588

[56] Zhu W Jiang Y. Determinants of caregiver burden of patients with haemorrhagic stroke in China. Int J Nurs Pract. (2019) 25:e12719. 10.1111/ijn.12719 30561838

[57] Pucciarelli G Lyons KS Petrizzo A Ambrosca R Simeone S Alvaro R. Protective role of caregiver preparedness on the relationship between depression and quality of life in stroke dyads. Stroke. (2021) 53:145–53. 10.1161/STROKEAHA.120.034029 34496626

[58] Ridley M Rao G Schilbach F Patel V. Poverty, depression, and anxiety: causal evidence and mechanisms. Science. (2020) 370:eaay0214. 10.3386/w27157 33303583

[59] Lopez V Copp G Molassiotis A. Male caregivers of patients with breast and gynecologic cancer: experiences from caring for their spouses and partners. Cancer Nurs. (2012) 35:402–10. 10.1097/NCC.0b013e318231daf0 22067685

[60] Atella V Piano Mortari A Kopinska J Belotti F Lapi F Cricelli C. Trends in age-related disease burden and healthcare utilization. Aging Cell. (2019) 18:e12861. 10.1111/acel.12861 30488641 PMC6351821

[61] Allen K Linn RT Gutierrez H Willer BS. Family burden following traumatic brain injury. Rehabil Psychol. (1994) 39:29. 10.1037/h0080313

[62] Li Q Lin Y Xu Y Zhou H. The impact of depression and anxiety on quality of life in Chinese cancer patient-family caregiver dyads, a cross-sectional study. Health Qual Life Outcomes. (2018) 16:230. 10.1186/s12955-018-1051-3 30545383 PMC6293618

[63] Götze H Brähler E Gansera L Schnabel A Gottschalk-Fleischer A Köhler N. Anxiety, depression and quality of life in family caregivers of palliative cancer patients during home care and after the patient's death. Eur J Cancer Care. (2018) 27:e12606. 10.1111/ecc.12606 27859889

[64] Koh YS Subramaniam M Matchar DB Hong SI Koh GC. The associations between caregivers' psychosocial characteristics and caregivers' depressive symptoms in stroke settings: a cohort study. BMC Psychol. (2022) 10:121. 10.1186/s40359-022-00828-2 35534900 PMC9082830

[65] Del-Pino-Casado R Rodríguez Cardosa M López-Martínez C Orgeta V. The association between subjective caregiver burden and depressive symptoms in carers of older relatives: a systematic review and meta-analysis. PLoS ONE. (2019) 14:e0217648. 10.1371/journal.pone.0217648 31141556 PMC6541277

[66] Collazzoni A Stratta P Pacitti F Rossi A Santarelli V Bustini M. Resilience as a mediator between interpersonal risk factors and hopelessness in depression. Front Psychiatry. (2020) 11:10. 10.3389/fpsyt.2020.00010 32184740 PMC7059212

[67] Ye Z Yang X Zeng C Wang Y Shen Z Li X. Resilience, social support, and coping as mediators between COVID-19-related stressful experiences and acute stress disorder among college students in China. Appl Psychol Health Well Being. (2020) 12:1074–94. 10.1111/aphw.12211 32666713 PMC7405224

[68] Dini M Poletti B Tagini S Reitano MR Allocco E Mazzocco K. Resilience, psychological well-being and daily functioning following hospitalization for respiratory distress due to SARS-CoV-2 infection. Healthcare. (2021) 9:1161. 10.3390/healthcare9091161 34574935 PMC8471260

[69] Curtis WJ Cicchetti D. Emotion and resilience: a multilevel investigation of hemispheric electroencephalogram asymmetry and emotion regulation in maltreated and nonmaltreated children. Dev Psychopathol. (2007) 19:811–40. 10.1017/S0954579407000405 17705904

[70] Masten AS. Ordinary magic. Resilience processes in development. Am Psychol. (2001) 56:227–38. 10.1037/0003-066X.56.3.227 11315249

[71] Charney DS. Psychobiological mechanisms of resilience and vulnerability: implications for successful adaptation to extreme stress. Am J Psychiatry. (2004) 161:195–216. 10.1176/appi.ajp.161.2.195 14754765

[72] Li J Chen YP Zhang J Lv MM Välimäki M Li YF. The mediating role of resilience and self-esteem between life events and coping styles among rural left-behind adolescents in China: a cross-sectional study. Front Psychiatry. (2020) 11:560556. 10.3389/fpsyt.2020.560556 33329099 PMC7714763

[73] Masten AS. Resilience in developing systems: progress and promise as the fourth wave rises. Dev Psychopathol. (2007) 19:921–30. 10.1017/S0954579407000442 17705908

[74] Lu Q Mårtensson J Zhao Y Johansson L. Needs of family members caring for stroke survivors in China: a deductive qualitative content analysis study by using the caregiver task inventory-25. BMC Geriatr. (2022) 22:96. 10.1186/s12877-022-02774-5 35114940 PMC8812361

[75] Üzar-Özçetin YS Hiçdurmaz D. Effects of an empowerment program on resilience and posttraumatic growth levels of cancer survivors: a randomized controlled feasibility trial. Cancer Nurs. (2019) 42:e1–13. 10.1097/NCC.0000000000000644 30256229

[76] Tsai SJ Li CC Tsai SM Kao SC Pai HC. The effect of action modules on resilience and psychological health of stroke patients: a pilot non-randomised control trial. J Clin Nurs. (2022). 10.1111/jocn.16238. [Epub ahead of print]. 35118746

[77] Cheng HY Chair SY Chau JPC. Effectiveness of a strength-oriented psychoeducation on caregiving competence, problem-solving abilities, psychosocial outcomes and physical health among family caregiver of stroke survivors: a randomised controlled trial. Int J Nurs Stud. (2018) 87:84–93. 10.1016/j.ijnurstu.2018.07.005 30059815

[78] Bakas T Austin JK Habermann B Jessup NM Mclennon SM Mitchell PH. Telephone assessment and skill-building kit for stroke caregivers: a randomized controlled clinical trial. Stroke. (2015) 46:3478–87. 10.1161/STROKEAHA.115.011099 26549488 PMC4659731

[79] Chuluunbaatar E Pu C Chou YJ. Changes in caregiver burden among informal caregivers of stroke patients in Mongolia. Top Stroke Rehabil. (2017) 24:314–21. 10.1080/10749357.2016.1277479 28095755

[80] Petursdottir AB Svavarsdottir EK. The effectivness of a strengths-oriented therapeutic conversation intervention on perceived support, well-being and burden among family caregivers in palliative home-care. J Adv Nurs. (2019) 75:3018–31. 10.1111/jan.14089 31162698

[81] Mackenzie C Kelly S Paton G Brady M Muir M. The living with dysarthria group for post-stroke dysarthria: the participant voice. Int J Lang Commun Disord. (2013) 48:402–20. 10.1111/1460-6984.12017 23889836

[82] Olson K Kemper KJ Mahan JD. What factors promote resilience and protect against burnout in first-year pediatric and medicine-pediatric residents? J Evid Based Complement Altern Med. (2015) 20:192–8. 10.1177/2156587214568894 25694128

[83] Hassani P Izadi-Avanji FS Rakhshan M Majd HA. A phenomenological study on resilience of the elderly suffering from chronic disease: a qualitative study. Psychol Res Behav Manag. (2017) 10:59–67. 10.2147/PRBM.S121336 28223851 PMC5304976

[84] Janssen BM Van Regenmortel T Abma TA. Identifying sources of strength: resilience from the perspective of older people receiving long-term community care. Eur J Ageing. (2011) 8:145–56. 10.1007/s10433-011-0190-8 21949496 PMC3156942

